# Beyond Moiré with Spatial Frequency Mastery via δ‐Function Expansion Metasurface

**DOI:** 10.1002/advs.202406819

**Published:** 2024-10-30

**Authors:** Rongsheng Chen, Feilong Yu, Jin Chen, Rong Jin, Jie Wang, Jiuxu Wang, Xiaoshuang Chen, Wei Lu, Guanhai Li

**Affiliations:** ^1^ State Key Laboratory of Infrared Physics Shanghai Institute of Technical Physics Chinese Academy of Sciences 500 Yu‐Tian Road Shanghai 200083 China; ^2^ School of Physical Science and Technology ShanghaiTech University Shanghai 201210 China; ^3^ University of Chinese Academy of Science No. 19A Yuquan Road Beijing 100049 China; ^4^ Hangzhou Institute for Advanced Study University of Chinese Academy of Sciences No.1 Sub‐Lane Xiangshan Hangzhou 310024 China; ^5^ Shanghai Research Center for Quantum Sciences 99 Xiupu Road Shanghai 201315 China

**Keywords:** δ‐function expansion, metasurface, moiré interference, optical calculation, spatial frequency modulation

## Abstract

Mastering spatial frequency manipulation within momentum space is pivotal yet challenging, particularly in mitigating moiré patterns that significantly impair image quality across diverse applications. Conventional methods often require trade‐offs in spatial resolution or fall short of completely eradicating unwanted frequencies, further burdened by complex post‐processing demands. In this work, a novel coherent δ‐function expansion technique implemented through an all‐silicon metasurface, affording unparalleled synergistic control over arbitrarily selected spatial frequencies via refined k‐space amplitude and phase modulations is introduced. This approach transcends traditional global methods by harnessing a sophisticated ensemble of multiple δ‐functions, enabling a holistic manipulation of spatial frequencies. The periodicity introduced by this approach also enables the feasibility of infinitely spatial stitching expansion for metasurfaces while maintaining high energy utilization efficiency. The methodology excels in the meticulous removal of local moiré frequencies while concurrently facilitating numerous advanced optical functions, including mixed partial differentiation and noise suppression, all within the optical domain. This work heralds a significant leap forward in optical manipulation, presenting a viable, scalable alternative to complex electronic post‐processing. Through this work, not only a longstanding challenge is addressed in optical physics but also open new avenues for research and application in photodetection and optical processing technologies.

## Graphical abstract

This work presents a novel approach to spatial frequency modulation using a coherent δ‐function expansion metasurface. By integrating polarization multiplexing and spatial control, the method achieves advanced optical functions such as moiré suppression, differentiation, and noise reduction. This scalable technique offers enhanced precision and versatility for optical information processing and photodetection applications.

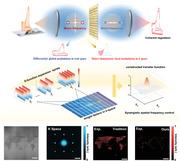

## 1 Introduction

The moiré phenomenon, an omnipresent interference pattern observed in layered materials from textiles to digital displays,^[^ ^1^ ^]^ poses a significant challenge in optical physics and information processing. This pattern, resulting from the superposition of two sine waves with closely matched spatial frequencies, produces complex arrays of shapes, spectral variations, and color intricacies. Such interference substantially compromises image quality, impacting crucial image processing tasks such as segmentation,^[^ ^2^ ^]^ feature extraction,^[^ ^3^ ^]^ and facial recognition,^[^ ^4^ ^]^ thereby hindering the fidelity of digital image capture and processing.^[^ ^5^ ^]^

Traditional methods to correct spatial frequency mismatches have largely depended on optical pre‐processing, including the use of low‐pass filters and modulating apertures,^[^ ^6^, ^7^, ^8^ ^]^ or post‐processing techniques with advanced interpolation algorithms.^[^ ^9^, ^10^, ^11^ ^]^ However, these strategies tend to uniformly affect the entire image, inadvertently eliminating high spatial frequency details vital for image resolution and feature detection.

The emergence of metasurfaces,^[^ ^12^ ^]^ capable of manipulating light at the subwavelength scale, marks a significant shift in optical control. Metasurfaces facilitate advanced light manipulation, such as vortex generation,^[^ ^13^, ^14^, ^15^ ^]^ the creation of exceptional points,^[^ ^16^, ^17^, ^18^ ^]^ and the execution of complex optical computations within momentum space,^[^ ^19^, ^20^, ^21^, ^22^, ^23^, ^24^, ^25^, ^26^, ^27^, ^28^, ^29^, ^30^ ^]^ multi‐dimensional control of polarization and wavelength^[^ ^31^, ^32^, ^33^ ^]^—achievements difficult with traditional materials. Yet, current implementations have taken a generalized approach to spatial frequency management, applying broad effects that overlook the intricate requirements of complex optical scenes.

In this work, we introduce a novel coherent δ‐function expansion method, employing an all‐silicon metasurface for precise engineering of specific spatial frequencies. This approach uses a strategic arrangement of metaatoms to tailor transfer functions, enabling advanced optical computations. We demonstrate the effectiveness of our method by fabricating and analyzing a “world map”, designed to selectively mitigate moiré patterns. Our technique's adept control over spatial displacement and orientation in momentum space facilitates a broad spectrum of optical calculations, including 1D and partial differentiation, and noise reduction, with polarization integration further enhancing multiplexing capabilities. Additionally, the periodic nature of this approach facilitates the possibility of extending the metasurface's size through spatial stitching, all while ensuring optimal energy utilization efficiency. Our work represents a significant advance in addressing moiré patterns, offering localized spatial frequency modulation without compromising optical resolution or resorting to complex computational interventions. It paves new pathways in image capture, information processing, and target recognition.

## 2 Results

To elucidate the efficacy of our coherent δ‐function expansion method for image processing within momentum space, we start with the image plane convolution integral:

(1) $$I\;\left(x_{i},y_{i}\right)=\int \;\;\;\int O\left(u,v\right)PSF\left(\frac{x_{i}}{M}-u,\frac{y_{i}}{M}-v\right)\;dudv$$

where *I*(*x_i_*,*y_i_*) denotes the image plane field, *O*(*u*, *v*) the object plane field, and *PSF* the point spread function. Given the *PSF* as the impulse function response, represented ideally by the δ function, we integrate the δ function in our analysis as follows^[^ ^34^ ^]^:

(2) $$\delta_{m}\left(x\right)=\frac{m}{\sqrt{\pi }}\exp\left(-m^{2}x^{2}\right)$$

Here, m serves as a weighting factor, with δ_*m*_ approximating the ideal δ function as m approaches infinity. The exponential function, capable of coherent expansion into components with opposing phases, facilitates the image processing operations—namely differentiation, integration, and filtering—through the superposition of multiple δ (exponential) functions.

For illustration, we apply our method to 1D first‐order differentiation. Based on its physical representation, the first‐order derivative of the *δ* function can be expressed as a combination of two δ functions with a π phase shift and displaced by a distance 2*s*_0_. Utilizing the Fourier transform property, we establish:

(3) $$\delta \left(x+s_{0}\right)-\delta \left(x-s_{0}\right)\overset{FT}{\Leftrightarrow }2i\;\sin\left(2\pi s_{0}k_{x}\right)=e^{i2\pi s_{0}k_{x}}-e^{-i2\pi s_{0}k_{x}}$$

Equation 3 facilitates the attainment of the targeted first‐order differential function by constructing the superposition of dual‐phase gradients within the momentum space (*k*‐space). This technique allows the construction of periodic transfer functions, thus enabling metasurfaces with unrestricted dimensions. It is worth noting that the spatial frequencies involved must exhibit harmonic relationships or, at the minimum, possess common factors. To further extend the generality of our method, we expand the transfer function *H* in series^[^ ^35^ ^]^:

(4) $$H\;\left(x\right)=\frac{a_{0}}{2}+\sum_{p=1}^{\infty }\left[a_{p}cos\left(\frac{p\pi x}{l}\right)+b_{p}sin\left(\frac{p\pi x}{l}\right)\right]\;$$

where $a_{p}=\frac{1}{l}\;\int_{-l}^{l}H\left(x\right)cos\left(\frac{p\pi x}{l}\right)dx$, $b_{p}=\frac{1}{l}\;\int_{-l}^{l}H\left(x\right)sin\left(\frac{p\pi x}{l}\right)dx$, *p* is an integer. This enables the synergistic construction of tailored transfer functions for any spatial frequency modulation by decomposing into multiple δ‐functions. In this situation, any arbitrarily selected spatial frequencies can be discriminately handled. Details can be found in Note S6 (Supporting Information).

The coherent δ‐function expansion method offers two primary advantages over previous techniques: First, the periodic or non‐periodic extension of δ functions within momentum space eliminates metasurface size constraints. Second, it enables the selective and synergistic engagement of distinct spatial frequencies for advanced optical operations, including differentiation, integration, and filtering, distinguishing this approach in the field of optical engineering.

In traditional metasurface designs, the modulation coefficients at specific positions in the Fourier plane are inherently coupled to the physical size of the metasurface. This coupling imposes significant limitations on the scalability of the system, which in turn constrains the practical applications. Our coherent δ‐function expansion method addresses this limitation by introducing a periodic structure that allows for the decoupling of the modulation coefficients from the metasurface size. Due to the periodic nature of the expansion, the system can be extended periodically, enabling it to modulate spatial frequencies across a larger area without increasing the physical size of the metasurface. This periodic extension provides a flexible framework for scaling up functionalities while maintaining the same compact physical footprint.

By decoupling the size constraints, our approach allows for the efficient integration of multiple functionalities, such as moiré pattern suppression and differentiation, within a single metasurface. This scalability makes our method highly adaptable for various applications that require high‐resolution spatial frequency manipulation, without the need for larger metasurfaces that would traditionally be required for such tasks.

**Figure 1:**
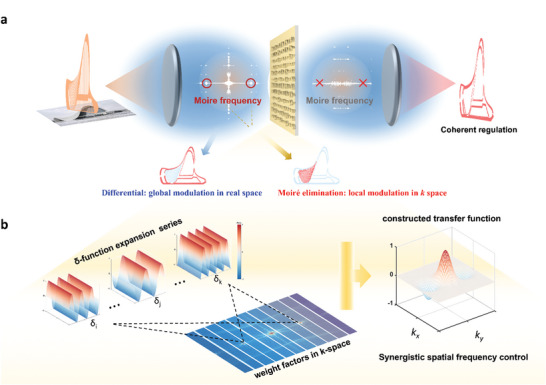
Synergistic Spatial Frequency Modulation with Coherent δ‐Function Expansion Metasurfaces. a) Illustration of an architectural image transforming a 4f optical system and a coherent δ‐function expansion metasurface, highlighting the integration of differentiation and moiré frequency suppression for enhanced image clarity with pronounced contours. This depiction contrasts traditional approaches where differentiation and moiré suppression, representing global and local computations respectively, are independently processed and often conflict in the frequency domain, limiting synergistic application. b) Outlines the foundational mechanism of the coherent δ‐function expansion method, where variable weight factors applied to δ functions within momentum space refine transfer functions. This adjustment facilitates the simultaneous execution of diverse global and local functions, enabling advanced control over spatial frequencies.

To illustrate the implementation of our coherent δ‐function expansion method, **Figure** **1** presents a schematic of how the metasurface operates within a 4f optical system, facilitating synergistic spatial frequency modulation. Figure 1a illustrates the coherent δ‐function expansion method applied to a metasurface, meticulously designed for effective spatial frequency modulation with an emphasis on eliminating moiré patterns. Utilizing the Heydar Aliyev Centre's architectural intricacies as an example, the figure demonstrates the isolation of moiré patterns through an initial Fourier transformation. This transformation, facilitated by the first lens in a 4f optical system, converts the image signal from real space to its frequency domain counterpart. Positioned at the Fourier plane, the metasurface dynamically modulates spatial frequencies in k‐space, enabling sophisticated optical computations. This includes blending differentiation with moiré pattern suppression, ultimately yielding a high‐resolution image with sharply defined contours, as processed by the second lens. Traditional methods often struggle to simultaneously achieve differentiation and moiré suppression due to overlapping frequency domain operations, thus limiting further optical computations. Traditional approaches to functionality implementation via frequency domain modulation typically involve direct mappings of individual functionalities. When attempting to combine multiple functionalities, these methods often result in a simplistic, patchwork‐like integration. This not only leads to mutual interference between functionalities but also limits further scalability. For example, when combining moiré pattern suppression with differentiation, a basic integration would result in reduced overall efficiency. In contrast, our approach leverages the point spread function (PSF) to reconstruct and seamlessly integrate multiple functionalities, enabling more effective combined operations and significantly enhancing scalability.

Figure 1b explores the underlying physics of the coherent δ‐function expansion method, which allows for the flexible modulation of selected spatial frequencies, harmoniously integrating differentiation with moiré frequency suppression. By employing expanded δ functions as Fourier coefficient factors, our technique precisely meets the modulation needs for various spatial frequencies. This dual function—global (optical differentiation) and local (moiré interference suppression) —is facilitated through δ function‐centric modulation. It effectively neutralizes the main spatial frequency components (when the transfer function H = 0) and eradicates high‐frequency elements responsible for moiré patterns. Observations behind the second lens reveal the complete removal of moiré patterns, surpassing traditional optical computation methods in efficacy.

**Figure 2:**
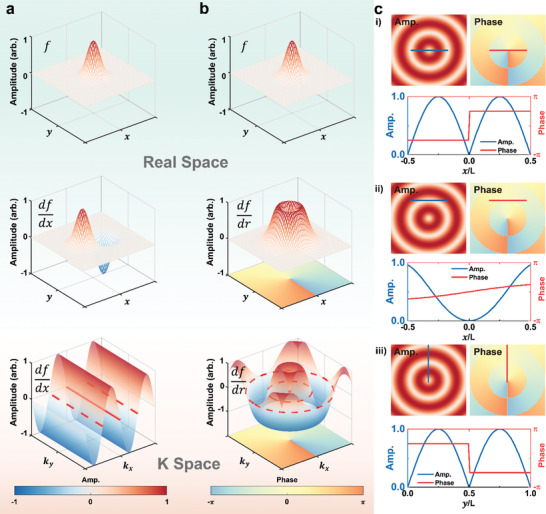
Transfer Function Development Through Coherent δ‐Function Expansion. a) The creation of a transfer function for 1D first‐order differentiation. It emphasizes the Fourier plane location (marked in red) where the transfer function H = 0, enables differentiation. b) The development of a transfer function for 2D first‐order differentiation, with a red indicator pointing to the crucial zero point of the transfer function, allows for effective 2D differentiation. c) A detailed look at the amplitude and phase modifications necessary for realizing the functionalities shown in (b), explaining how the transfer function accommodates different differentiation operations at certain k‐space locations. From top i) to bottom iii), panels display the execution of first‐order differentiation along the x‐axis, second‐order differentiation along the same axis, and first‐order differentiation along the y‐axis.

To illuminate the design principles of our novel approach, we illustrate the development of diverse transfer functions via synergistic spatial frequency modulations using the coherent δ‐function expansion method. **Figure** **2** further explores the transfer functions created through this method, showcasing the versatility of the δ‐function expansion in achieving 1D and 2D differentiation by modulating spatial frequencies. Figure 2a displays the normalized amplitude profiles of the δ function in its initial and differentiated states in real space (top and middle panels, respectively), along with their Fourier transformations in the momentum domain (bottom panel). The red lines indicate where $\tilde{H}\left(x\right)\approx 0$, setting the stage for optical differentiation. Building on this concept, Figure 2b explores the implementation of 2D first‐order differentiation, incorporating amplitude modulation in both dimensions and introducing a vortex phase with a topological charge of + 1. The red dashed rings, mirroring the lines in Figure 2a, highlight regions of selective spatial frequency modulation, pinpointing areas where optical differentiation is most effective. In our method, a vortex phase with a topological charge of 1 is introduced to achieve precise 2D differentiation by inducing a controlled π phase shift between positive and negative spatial frequency components. This ensures that the transfer function *H*  = *k_x_* , maintains equal amplitude values while introducing the necessary phase difference for accurate differentiation. The vortex phase enables seamless modulation across both axes in the frequency domain, allowing for enhanced edge detection and feature extraction in 2D images. This method extends beyond traditional 1D differentiation by facilitating selective modulation of spatial frequencies, improving optical computations for higher‐dimensional tasks. Previous work, such as the use of vortex phase plates for 2D edge extraction,^[^ ^22^ ^]^ supports the effectiveness of this approach. Integrating the vortex phase into our method provides more precise and flexible differentiation, essential for complex imaging and feature enhancement tasks.

Figure 2c showcases the multiplexing potential of the coherent δ‐function expansion approach. Through precise adjustments of the metasurface's placement within k‐space, we unlock a range of transfer functions. The series displayed—from top to bottom—demonstrates the organized employment of a single metasurface to execute first‐order differentiation along the x‐axis, second‐order differentiation along the same axis, and first‐order differentiation along the y‐axis. This flexibility underlines the method's ability to synergistically perform either the same or distinct operations on signals of various spatial frequencies or to achieve a plethora of functionalities through careful metasurface alignment and orientation.

**Figure 3:**
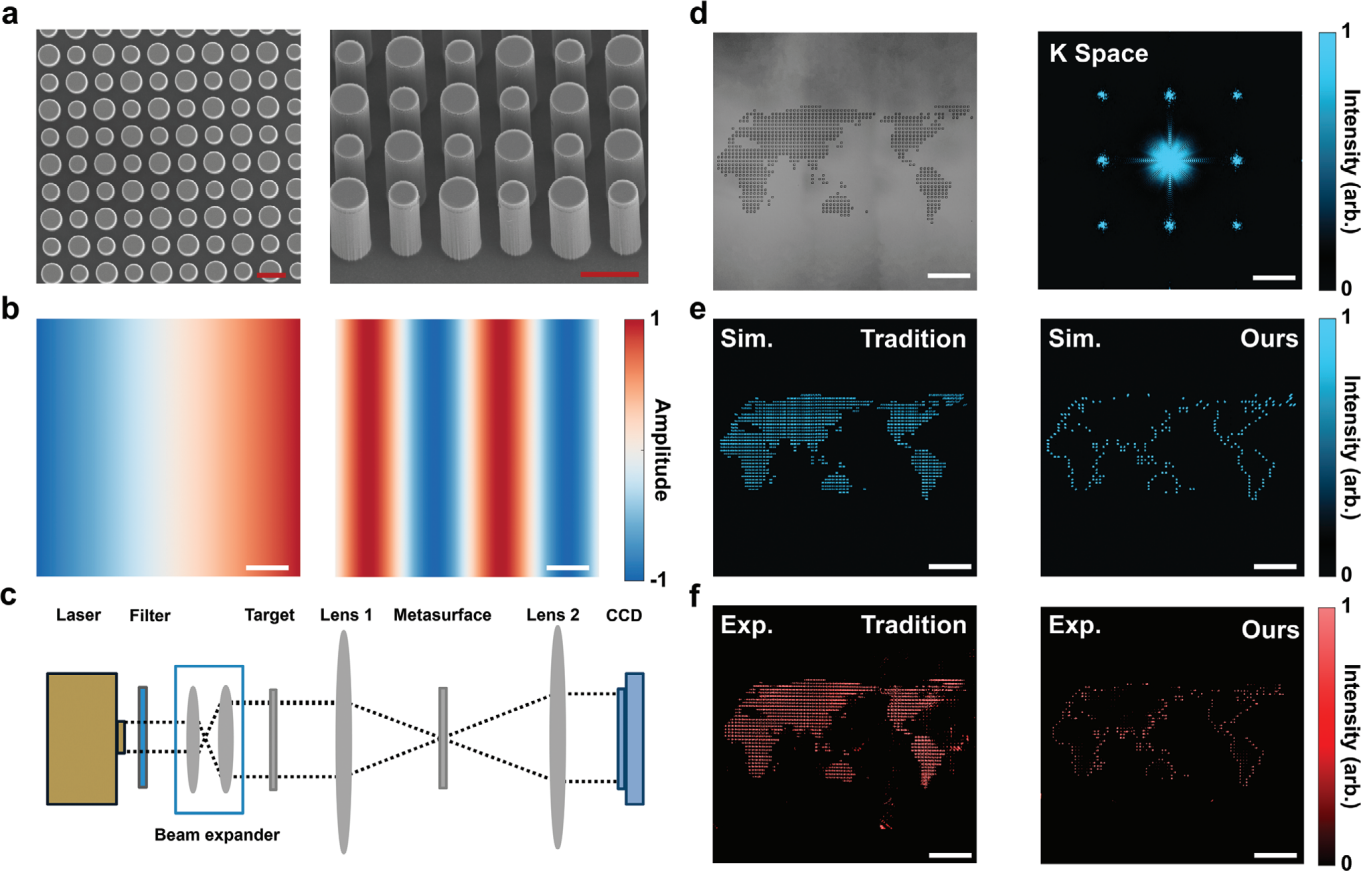
Moiré Pattern Suppression via Spatial Frequency Modulation. a) SEM images display top and side views of the intricately etched metasurface, showcasing its detailed structure. b) Comparison of amplitude modulation techniques for first‐order differentiation between traditional linear modulation (left) and our coherent δ‐function expansion method (right). c) The schematical experiment setup. It details the light's journey through the system to demonstrate the operational dynamics. d) The visible light microscopy image of the specially designed phase‐only imaging target, including its spatial frequency distribution within the Fourier plane. e) Contrasts simulated results of metasurface designs: the conventional linear modulation approach (left) against the innovative coherent δ‐function expansion (right). f) Experimental results mirror the simulations in (e), with the left image from the standard linear method and the right image from our δ‐function approach. Red scale bar: 2 µm; white scale bar: 500 µm.

To demonstrate the practical effectiveness of our approach, **Figure** **3** compares the results of traditional methods with our coherent δ‐function expansion method, highlighting the superior suppression of high‐frequency noise and elimination of moiré patterns. To validate its effectiveness in mitigating moiré fringes, we fabricated an all‐silicon metasurface. This metasurface, intricately etched from a single‐crystalline silicon substrate, is composed of cylindrical metaatoms arranged at a periodicity of 1.7 µm and standing 3 µm tall. Optimized for a 4 µm wavelength within the mid‐wavelength infrared spectrum, it spans a 3 mm by 3 mm area. Figure 3a displays scanning electron microscopy (SEM) images of the metasurface from top and side perspectives, illustrating the advanced amplitude and phase modulation capabilities facilitated by the metaatoms’ interactions. This configuration specifically targets the suppression of spatial frequencies similar to those produced in a “world map” sample, employed here as a model for moiré fringe generation using dual gratings.

In Figure 3b, a clear distinction is drawn between the transfer functions achievable through our δ‐function expansion method and those attainable by traditional means. Whereas conventional approaches typically manage image differentiation at a single zero position where $\tilde{H}\;\left(x\right)=0$ at x = 0, our strategy introduces multiple zero positions to fulfill the $\tilde{H}\;\left(x\right)=0$ condition. This periodic zeroing provides a unique advantage in eliminating non‐zero k‐space information, thereby allowing our technique to selectively target undesirable spatial frequencies.

The experimental setup, illustrated in Figure 3c, integrates the metasurface within a 4f optical system, precisely modulating the transmitted light's spatial frequencies in momentum space. Figure 3d highlights a “world map” imaging target, consisting of dot matrices on a Si substrate designed to emulate moiré patterns and emphasizing the π phase difference between the colored dots and their background. Detailed design specifications are available in Note S5 (Supporting Information). This arrangement not only clarifies the target's spatial frequencies in k‐space but also showcases the effectiveness of first‐order differential amplitude modulations along the x‐axis, a capability attributed to our metasurface's novel design. The simulation and experimental results, depicted in Figures 3e,f, respectively, vividly demonstrate our coherent δ‐function expansion method's superiority over traditional approaches in reducing high‐frequency noise and eliminating moiré fringes. The close alignment of experimental findings with simulations further affirms our method's efficiency. Regarding the observed edge loss, this is due to the specific metasurface design used for the demonstration, which adopts a transfer function that varies only along the x‐direction. As a result, it can effectively extract edges that are not strictly aligned with the x‐direction, but it may not capture edges that are exactly aligned with it.

**Figure 4:**
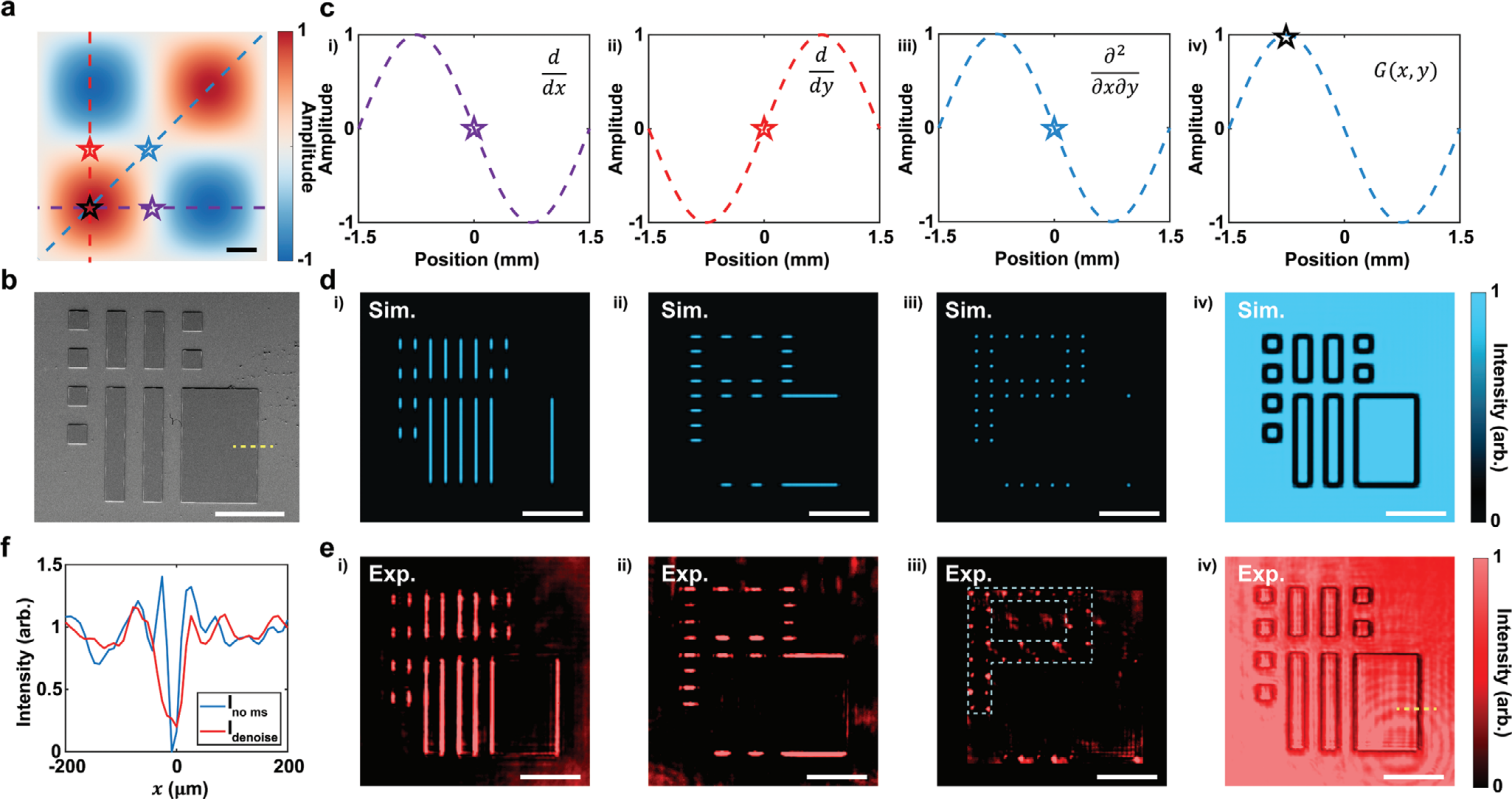
Multifunctional Spatial Frequency Control Using a Single Metasurface. a) The amplitude modulation is achieved through the coherent δ‐function expansion method, where amplitude shifts (positive or negative) imply a π phase change. Different colored lines highlight the tailored phase and amplitude modulations at various orientations. b) SEM images of the custom multifunctional phase target, reveal the complexity of its structure. c) The amplitude variations along specific lines, indicated by hollow pentagrams at optical axis intersections, demonstrate: i) first‐order differentiation along the x‐axis; ii) first‐order differentiation along the y‐axis; iii) mixed partial differentiation for vertex extraction; and iv) precise noise reduction. d) Simulated output images showing the metasurface's alignment with the target and its execution of diverse spatial frequency modulations in momentum space, effectively realizing the functionalities presented in (c). e) Measured images confirming the metasurface's alignment and implementation of various spatial frequency modulations in momentum space, correlating with simulation results. f) Illustrates noise reduction efficiency, with red and blue curves representing intensity profiles along yellow dashed lines in (b) and (e iii). Scale bar: 500 µm.

**Figure** **4** expands on this by showcasing how the metasurface's spatial positioning and orientation within k‐space can be adjusted to enable a range of functionalities, including differentiation and noise reduction. Figure 4 elucidates a series of functionalities by manipulating the metasurface's spatial positioning and orientation within k‐space. It enables the generation of a variety of transfer functions, spanning mixed partial differentiation to noise suppression, with comprehensive details available in Notes S2–S4 (Supporting Information). Figure 4a underlines the dependence of transfer functions on the precise alignment of the metasurface within the momentum space of a 4f optical system, highlighted by four pentagonal stars marking specific positions and orientations along the optical axis.

Figure 4b presents direct imaging outcomes from the 4f system, showcasing a bespoke target of strip patterns etched into the silicon substrate. Figure 4c depicts amplitude modulations linked to the colored tangents in Figure 4a, targeting functionalities such as first‐order differentiation along the x/y directions, vertex extraction, and noise reduction.

The simulation and experimental results are illustrated in Figures 4d,e. Subplots i and ii display the achievement of single‐line edge extractions in specified directions, demonstrating adept first‐order differential operations. Subplot iii reveals the disappearance of the original shape, unmasking a “P” formed from a dot array—evidence of vertex extraction through mixed partial differentiation. A dashed semi‐transparent line in the experimental image outlines the original shape, emphasizing the method's foundation on four δ functions that conjointly compose a 2 × 2 operator, mathematically represented by the matrix: $\left(\begin{array}{cc} 1 & -1\\ -1 & 1 \end{array}\right)$, where the negative sign denotes a phase shift of π. Subplot iv showcases the noise reduction achieved, with Figure 4f comparing intensity profiles along the yellow lines in Figures 4b,e. The observed post‐denoising effect, marked by increased line thickness and reduced depth, signifies effective noise suppression. This demonstration of our method's capacity for intricate spatial frequency engineering within k‐space, achieving functionalities previously unreachable, highlights its great potential in optical processing and imaging technologies.

**Figure 5:**
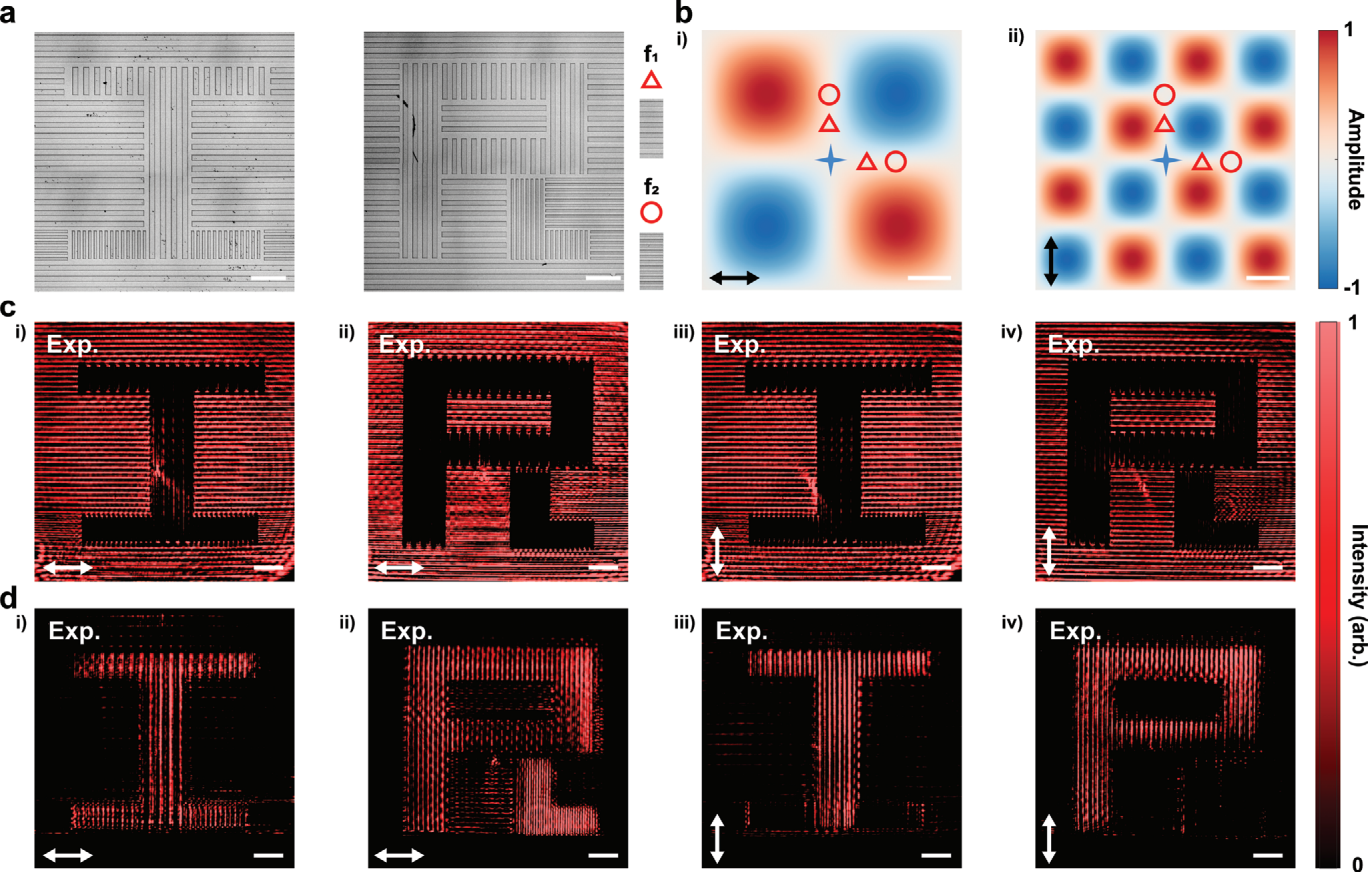
Polarization and Spatial Multiplexing via Coherent δ‐Function Expansion. a) The visible light microscopy images of two‐phase grating targets are tailored for this spatial frequency modulation. b) shows the amplitude modulation and spatial frequency mapping for i) x‐polarized and ii) y‐polarized light, pinpointing the metasurface's strategic positioning within the Fourier plane as essential. The blue cross denotes the metasurface's central location, while hollow triangles and circles represent the Fourier plane projections of the targets’ varied spatial frequencies. c)The polarization‐independent imaging results following a lateral displacement of the metasurface by 1/8 of its side length, emphasizing consistent imaging across different polarization states. d) Illustration of polarization‐dependent imaging results after a longitudinal displacement of the metasurface by 1/8 of its side length: i, ii) for x‐polarization, iii, iv) for y‐polarization. Scale bar: 700 µm.

Finally, **Figure** **5** demonstrates the polarization‐dependent imaging results, emphasizing the distinct effects of lateral and longitudinal metasurface displacements on polarization response. Our coherent δ‐function expansion method's adaptability in handling a wide range of spatial frequencies within momentum space is further exemplified by the integration of polarization multiplexing, as illustrated in Figure 5. Figure 5a presents two distinct imaging targets, each composed of binary phase gratings with 0/π phase shifts, encoding unique vectorial spatial frequencies, *f*_1_ and *f*_2_, with further details provided in Note S5 (Supporting Information). Figure 5b elaborates on the metasurface's transfer functions when interacting with i) x‐ and ii) y‐polarized light. For both polarizations, the metasurface facilitates mixed partial differential operations. Central stars denote the metasurface's operational axis within the 4f system, while triangles and circles represent the orientations of different gratings in k‐space, leading to a periodic normalized amplitude distribution where the spatial frequency along the y‐axis surpasses that along the x‐axis—a notable departure from traditional setups.

Figure 5c displays imaging outcomes with the metasurface laterally shifted by an eighth of its side length, with subplots i, iii) about target 1, and ii, iv) to target 2, as indicated by white arrows showing the polarization directions. This lateral displacement results in consistent imaging effects across different polarizations for the same target, producing intaglio renditions of the letters “I” or “R”. Conversely, Figure 5d shows the imaging results with the metasurface longitudinally displaced by an eighth of its side length, contrasting with Figure 5c's findings by offering distinct imaging outcomes under different polarizations. Specifically, x‐polarization reveals the letters “I” and “R”, whereas y‐polarization exposes “T” and “P”. This demonstrates the method's nuanced control over spatial frequencies associated with varying displacements, achieving distinct imaging effects in real space. The distinct polarization responses under different metasurface displacement directions arise from the nature of the interaction between the incident light and the metasurface structure. During lateral displacement, the metasurface structure interacts with the incoming light symmetrically with respect to polarization. Both x‐ and y‐polarized light are similarly modulated, resulting in minimal polarization effects in the imaging output. In contrast, during longitudinal displacement, the interaction is asymmetric, allowing the metasurface to selectively modulate the phase and amplitude of different polarization components. This design leads to distinct polarization‐dependent responses, where x‐ and y‐polarized light are modulated differently, producing varying imaging outcomes depending on the polarization state. This directional dependence of the polarization response highlights the versatility of our metasurface design in controlling light properties, particularly under varying displacement conditions. This showcase underscores our technique's unparalleled capability to modulate spatial frequencies via polarization‐dependent displacement, introducing a novel dimension of versatility in optical processing and paving the way for sophisticated imaging applications. In **Table** **1**, we further compared our Moiré pattern removal scheme and optical computing metasurface scheme with previously reported ones, demonstrating our advantages. The comparison includes the scalability while maintaining high efficiency, the implementation of multiple functions, and the multiplexing channel numbers. The four positions in the multiplexing channels of Table 1 refer to distinct functionalities that our metasurface design can achieve under various spatial configurations, such as noise reduction, mixed partial differentiation, and first‐order differentiation along the x and y directions.

**Table 1:** Comparison between our work and reported schemes for Moiré removal and optical computing.

References	Size scalability	Multiple Functions			Multiplexing Channels	
		Optical computing [k=0]	Moiré removal [k≠0]	simultaneous work？	Polarization	Space
Low‐pass filter in cameras Canon EOS R5, Sony Alpha 7 IV	NA	No	Yes	No	1	No
Ref. [19]	No	Yes	No		1	No
Ref. [22]					2	
Ref. [36]					1	
Ref. [33]	NA				1	3 angles
Our work	Yes	Yes	Yes	Yes	2	4 positions

## 3 Discussion and Conclusion

Our study introduces a promising approach in optical processing through the coherent δ‐function expansion method, marking a significant leap forward in the synergistic manipulation of local and arbitrarily selected spatial frequencies within momentum space. This method not only addresses the longstanding challenge of moiré pattern suppression but also demonstrates versatility in synergistically performing a wide array of optical computations, from differentiation and integration to noise reduction. The use of silicon metasurfaces, intricately designed with cylindrical metaatoms, enables precise control over the amplitude and phase of transmitted light, thereby facilitating the selective manipulation of spatial frequencies. Our method's ability to enable precise local control over selectively chosen spatial frequencies through *k*‐space amplitude and phase modulations relying on a complex overlay of multiple δ‐functions represents a departure from global strategies.

The experimental outcomes underscore the method's versatility, not only in the thorough removal of moiré patterns but also in facilitating various optical functions such as mixed partial differentiation and noise suppression within the optical domain. This is achieved without compromising image resolution, thus overcoming a significant limitation of previous approaches. The integration of polarization multiplexing further exemplifies the method's adaptability, offering nuanced control over spatial frequencies and enabling a novel dimension of versatility in optical processing. The introduction of polarization multiplexing further exemplifies the method's adaptability, allowing for the flexible manipulation of different spatial frequencies and the achievement of distinct imaging outcomes through lateral and longitudinal metasurface displacements. This nuanced control over spatial frequencies paves the way for new avenues in optical processing, where the specific requirements of an application can dictate the manipulation strategy employed.

In conclusion, the coherent δ‐function expansion method via an all‐silicon metasurface presents a substantial advancement in the field of optical manipulation. By enabling synergistic control over arbitrarily selected spatial frequencies through k‐space amplitude and phase modulations, this approach significantly enhances the ability to mitigate moiré patterns and execute advanced optical functions. The implications of this work extend beyond the immediate resolution of moiré patterns, promising to push forward the imaging, photodetection, and optical processing technologies.

## 4 Experimental Section

### Sampling Fabrication

The fabrication of the all‐silicon metasurface began with the deposition of a 50 nm chromium (Cr) layer onto a double‐sided polished silicon wafer via electron beam evaporation. This was followed by spin‐coating a photoresist layer onto the Cr‐coated wafer, which was then baked on a hotplate to achieve optimal adhesion and uniformity. The complex metasurface pattern was delineated using advanced electron beam lithography with a JBX‐6300FS system. Post‐lithography, the sample was developed in a 300‐MIF developer, rinsed thoroughly with deionized water, and dried. The fabrication culminated with inductively coupled plasma dry etching of both Cr and silicon layers, utilizing selective gas chemistry to etch the desired metasurface architecture precisely.

### Experimental Setup

Referenced in Figure 3c of the manuscript, the optical system employed a supercontinuum laser (Leukos Electro MIR 4.8), with its output refined through a 4 µm spectral filter. A beam expander, composed of two lenses (15 and 75 mm focal lengths), broadened the laser beam to encompass the intended imaging area. The imaging target was situated on the object plane of a 4*f* system, which incorporates lenses of focal lengths *f*_1_ =  15 mm and *f*_2_ =  25.4 mm, with the metasurface positioned at the system's Fourier plane. Detection was performed by a homemade cooled mid‐wave infrared camera, featuring a 640 × 512 resolution and 15 µm pixel size.

### Metasurface Design and Simulations

Initially, metaatom characteristics were assessed using rigorous coupled wave analysis (RCWA) to create a comprehensive database. The metasurface's amplitude and phase modulation efficacy were verified through finite‐difference time‐domain (FDTD) simulations (Ansys Lumerical FDTD). In subsequent optimizations, square “four‐in‐one” metaatom configurations were utilized, denoted as $\left(\begin{array}{cc} A & B\\ B & A \end{array}\right)$, enabling direct amplitude‐phase characteristic evaluation. This phase involved considering the supercell's periodicity and pillar height, alongside the dimensions of the long and short axes of each pillar, resulting in four critical variables. The comprehensive database for metasurface construction was compiled using a Python‐based RCWA script with GPU acceleration, executed on a server equipped with dual NVIDIA RTX 3090 graphics cards, requiring ≈ 2 weeks to complete a dense and varied unit database.

### Data Availability

Relevant data supporting the key findings of this study are available in the article and Supplementary Information file. All raw data generated in this study are available from the corresponding authors upon reasonable request.

### Code Availability

The code used for data analysis during this study is available upon reasonable request from the corresponding author.

## Conflict of Interest

The authors declare no conflict of interest.

## Supporting information



Supporting Information

## Data Availability

The data that support the findings of this study are available from the corresponding author upon reasonable request.
