# Supplementary material for: Beyond Moiré with Spatial Frequency Mastery via δ‐Function Expansion Metasurface
Source: Adv Sci (Weinh). 2024 Oct 30;11(47):2406819. doi: 10.1002/advs.202406819 (PMC11653613; doi:10.1002/advs.202406819)
Supplement: Supplementary file 1 — Supporting Information [file ADVS-11-2406819-s001.docx]

[https://doi.org/10.1002/advs.202408030https://doi.org/10.1002/advs.202408030](https://doi.org/10.1002/advs.202408030)Supplementary Materials for

**Beyond Moiré with Spatial Frequency Mastery via δ-Function Expansion Metasurface**

**Rongsheng Chen^1,2,3#^, Feilong Yu^1#^, Jin Chen^1#^, Rong Jin^1,3^, Jie Wang^1^, Jiuxu Wang^1,3^, Xiaoshuang Chen^1,3,4,5^, Wei Lu^1,2,3,4,5^, Guanhai Li^1,3,4,5*^**

^1^State Key Laboratory of Infrared Physics, Shanghai Institute of Technical Physics, Chinese Academy of Sciences, 500 Yu-Tian Road, Shanghai, 200083, China

^2^School of Physical Science and Technology, ShanghaiTech University, Shanghai 201210, China

^3^University of Chinese Academy of Science, No. 19A Yuquan Road, Beijing 100049, China

^4^Hangzhou Institute for Advanced Study, University of Chinese Academy of Sciences, No.1 Sub-Lane Xiangshan, Hangzhou, 310024, China

^5^Shanghai Research Center for Quantum Sciences, 99 Xiupu Road, Shanghai, 201315, China

* Corresponding Email: ghli0120@mail.sitp.ac.cn

Note 1: Derivation of the transfer function for *n*th-order differentiation operation with conventional approaches

Traditionally, the transfer function is obtained by directly operates the function as a whole, which means that the transfer function corresponding to a single function needs to occupy the entire controllable frequency domain space. For example, for $n$th order differential operations along $x$-direction, the transfer function in $k$-space can be derived under traditional schemes based on the differential property of Fourier trasnform^1^,

$$\begin{aligned} \tilde{H}\left( k_{x_{1}} \right)\propto\left( e^{i\frac{\pi}{2}}k_{x_{1}} \right)^{n}\#\left( S AUTONUM \backslash* Arabic \right) \end{aligned}$$

It should be noted that in the 4-$f$ system, the information of space frequency $k_{x_{1}}$ was transferred into space coordinates information at Fourier plane, so if there is such a transfer function on the Fourier plane (real space), differentiation of arbitrary order can be achieved by optical systems and this conclusion can be generalized to some other operations. Considering that gain is usually not introduced in such optical systems, modulation can only be achieved through normalization. In traditional first-order differentiation, the metasurface requires linear modulation with the transmittance of 0 in the central position and the transmittance of 1 at the edge, and the phase difference of $\pi$ is added to the +/- range of the $x$-coordinate.


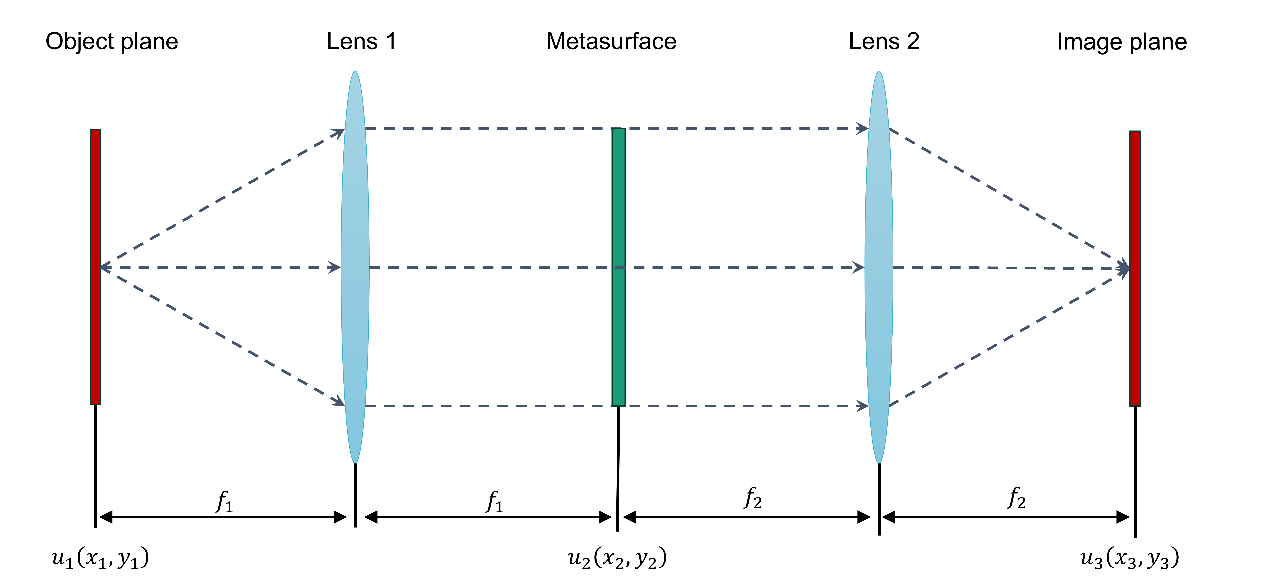


**Fig. S1: Optical path diagram for the derivation process in conventional approaches.**

Note 2: Derivation of one-dimension first-order differentiation operation

In the imaging system as showed in **Fig. S1**, the final output is the convolution of the incident image and the point spread function of the system

$$\begin{aligned} E_{out}=E_{in}\bigotimes PSF\#\left( S AUTONUM \backslash* Arabic \right) \end{aligned}$$

where $E_{in}$ and $E_{out}$ are the input and output images, $PSF$ is the point spread function of the system, which is the $\delta$ function ideally. Due to the following properties of convolution

$$\begin{aligned} F\left( E_{out} \right)=F\left( E_{in}\bigotimes PSF \right)=E_{in}\bigotimes F\left( PSF \right)\#\left( S AUTONUM \backslash* Arabic \right) \end{aligned}$$

where $F$ represents operations such as integration and differentiation. Therefore, the operation on the image can be transferred to $PSF$, then the Fourier transform can be performed to obtain the required k-space modulation. The difference between the previously reported works and ours is that we utilize the properties of the $\delta$ function to perform optical operations through modulation methods that can be periodically extended.

A definition of the $\delta$ function is shown in the following equation^2^

$$\begin{aligned} \delta_{m}\left( x_{1} \right)=\frac{m}{\sqrt{\pi}}exp\left( -m^{2}x_{1}^{2} \right)\#\left( S AUTONUM \backslash* Arabic \right) \end{aligned}$$

when $m$ is close to ∞, $\delta_{m}\left( x \right)$ is the traditional $\delta$ function. Based on this definition, the one-dimension first-order differential form of the $\delta$ function is

$$\begin{aligned} \frac{{d\delta}_{m}\left( x_{1} \right)}{dx}=\frac{-2m^{3}x_{1}}{\sqrt{\pi}}exp\left( -m^{2}x_{1}^{2} \right)\#\left( S AUTONUM \backslash* Arabic \right) \end{aligned}$$

the form of first-order differential of a $\delta$ function is two $\delta$ functions with $\pi$ phase difference staggered by a certain distance. Since the following Fourier transform property,

$$\begin{aligned} \delta\left( x_{1}+s_{0} \right)-\delta\left( x_{1}-s_{0} \right)\underset{\Leftrightarrow}{FT}2i\sin\left( 2\pi s_{0}k_{x_{1}} \right)=e^{i2\pi s_{0}k_{x_{1}}}-e^{-i2\pi s_{0}k_{x_{1}}}\#\left( S AUTONUM \backslash* Arabic \right) \end{aligned}$$

the needed modulation of metasurface at Fourier plane is

$$H_{1d1st}(x_{2})\propto e^{i2\pi s_{0}x_{2}}-e^{-i2\pi s_{0}x_{2}}$$

The superposition of the dual phase gradients at the Fourier plane can be employed to achieve the desired operation.

Note 3: Derivation of one-dimension second-order differentiation operation

The one-dimension second-order differential form of the $\delta$ function is

$$\begin{aligned} \frac{{d^{2}\delta}_{m}\left( x_{1} \right)}{dx^{2}}=\frac{2m^{3}}{\sqrt{\pi}}({2m^{2}x_{1}^{2}-1)e}^{-m^{2}x_{1}^{2}}\#\left( S AUTONUM \backslash* Arabic \right) \end{aligned}$$

The corresponding physical images are three $\delta$ functions, and the amplitude ratio of which is (-1):2:(-1), where the positive and negative signs represent the phase difference of $\pi$. The middle double amplitude term can be regarded as two $\delta$ functions, and because of the following Fourier transform pairs:

$$\begin{aligned} 2\delta\left( x_{1} \right)-\delta\left( x_{1}+s_{0} \right)-\delta\left( x_{1}-s_{0} \right)\underset{\Leftrightarrow}{FT}2-2\cos\left( 2\pi s_{0}k_{x_{1}} \right)=2-e^{i2\pi s_{0}k_{x_{1}}}-e^{-i2\pi s_{0}k_{x_{1}}}\#\left( S AUTONUM \backslash* Arabic \right) \end{aligned}$$

four sets of pure phase units with the following modulation $H_{1d2nd}(x)$ can be used to achieve second-order derivative regulation in $k$-space, which is also periodic and can be extended infinitely.

$$\begin{aligned} H_{1d2nd}\left( x_{2} \right)\propto2e^{i0}-e^{i2\pi s_{0}x_{2}}-e^{-i2\pi s_{0}x_{2}}\#\left( S AUTONUM \backslash* Arabic \right) \end{aligned}$$

the superposition of these gradients at Fourier plane can be used to achieve the expected operation.


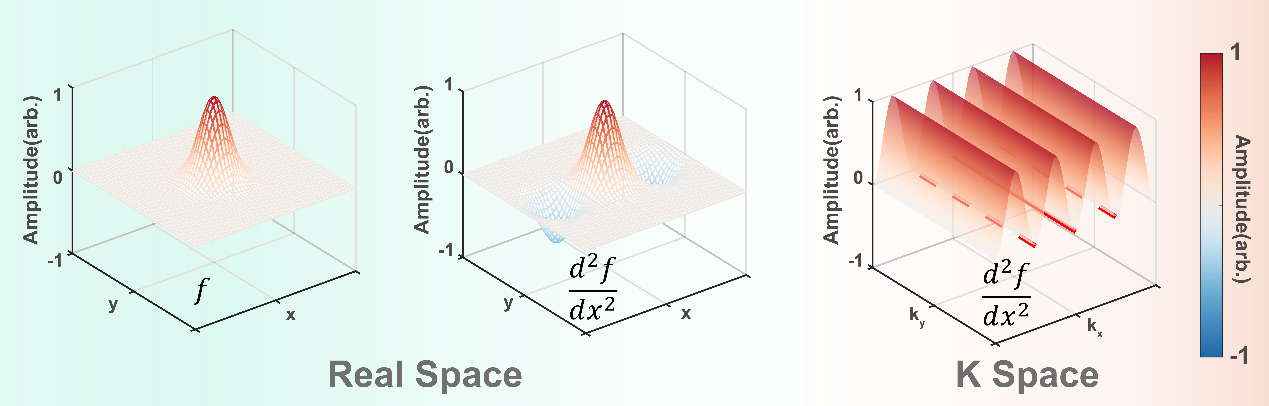


**Fig. S2: Schematic derivation of the transfer function for one-dimension second-order differentiation operation** **with coherent** $\boldsymbol{\delta}$**-function expansion approach.**

Note 4: Derivation of mixed partial differentiation operation

Mixed partial derivatives are operations that extract vertices of images in two orthogonal directions (such as $x$ and $y$ directions), and they can also perform edge detection for oblique edges. The operator of mixed partial derivatives is $\frac{\partial^{2}}{\partial x_{1}\partial y_{1}}$, and unlike the previously mentioned two-dimensional first-order and second-order derivatives, this operator is not isotropic. The expression of the $\delta$ function in two dimensions is

$$\begin{aligned} \delta_{m}\left( x_{1},y_{1} \right)=\frac{m}{\sqrt{\pi}}e^{-m^{2}\left( x_{1}^{2}+y_{1}^{2} \right)}\#\left( S AUTONUM \backslash* Arabic \right) \end{aligned}$$

and the result of its mixed partial derivative is

$$\begin{aligned} \frac{\partial^{2}\delta_{m}\left( x_{1},y_{1} \right)}{\partial x_{1}\partial y_{1}}=\frac{4m^{5}}{\sqrt{\pi}}x_{1}y_{1}e^{-m^{2}\left( x_{1}^{2}+y_{1}^{2} \right)}\#\left( S AUTONUM \backslash* Arabic \right) \end{aligned}$$

The corresponding physical image is four $\delta$ functions, forming a 2×2 lattice, whose arrangement can be represented by the following matrix:

$$\left( \begin{matrix} 1 & -1 \\ -1 & 1 \end{matrix} \right)$$

The sign indicates the phase difference of $\pi$. Similarly, because of the following Fourier transform pair:

$$\begin{aligned} \delta\left( x_{1}-s_{0},y_{1}-s_{0} \right)+\delta\left( x_{1}+s_{0},y_{1}+s_{0} \right)-\delta\left( x_{1}+s_{0},y_{1}-s_{0} \right)-\delta\left( x_{1}-s_{0},y_{1}+s_{0} \right) \\ \underset{\Leftrightarrow}{FT}2\cos\left( 2\pi s_{0}\left( k_{x_{1}}+k_{y_{1}} \right) \right)-2\cos\left( 2\pi s_{0}\left( k_{x_{1}}-k_{y_{1}} \right) \right) \\ =e^{i2\pi s_{0}\left( k_{x_{1}}+k_{y_{1}} \right)}+e^{-i2\pi s_{0}\left( k_{x_{1}}+k_{y_{1}} \right)}-e^{i2\pi s_{0}\left( k_{x_{1}}-k_{y_{1}} \right)}-e^{-i2\pi s_{0}\left( k_{x_{1}}-k_{y_{1}} \right)}\#\left( S AUTONUM \backslash* Arabic \right) \end{aligned}$$

Therefore, by the same logic, pure phase elements can be used to implement the amplitude modulation in the following equation in $k$-space or on the Fourier plane, achieving the mixed partial derivative effect.

$$\begin{aligned} H_{partial dev}\left( x_{2},y_{2} \right)\propto e^{i2\pi s_{0}\left( x_{2}+y_{2} \right)}+e^{-i2\pi s_{0}\left( x_{2}+y_{2} \right)}-e^{i2\pi s_{0}\left( x_{2}-y_{2} \right)}-e^{-i2\pi s_{0}\left( x_{2}-y_{2} \right)}\#\left( S AUTONUM \backslash* Arabic \right) \end{aligned}$$

Note 5: The parameters of the fabricated imaging targets

The imaging targets used in the experiment are all pure Si mid-infrared phase targets. The map target used in **Fig. 3** has a two-dimensional grating period of $d=80$ µm, a square side length of $a=40$ µm, and an etching height of 3 µm. The letter grating target used in **Fig. 5** has two types of one-dimensional gratings, whose parameters are (1) $d=80$ µm, $a=b=40$ µm, and (2) $d=160$ µm, $a=b=80$µm. The two sets of gratings have a frequency ratio of 2. Due to the large size of the target, its length of long edge > 3 mm, so the optical microscope images in the paper are stitched from multiple images. In the target used in **Fig. 4** of the main text, the minimum side length is 120 µm, the minimum spacing is 120 µm, and the etching height is 5 µm.

Note 6: Construction of tailored transfer functions for any spatial frequency modulation with the coherent $\boldsymbol{\delta}$-function expansion

If the controllable region of the metasurface has a length of $2l$ in the $x$ direction, its modulation function $H\left( x \right)$ is a periodic function with a period of $2l$ on [$-l$, $l$], satisfying the Dirichlet convergence condition, and can be expanded into a Fourier series.

$$\begin{aligned} H\left( x_{2} \right)=\frac{a_{0}}{2}+\sum_{p=1}^{\infty} \left[ a_{p}cos\left( \frac{p\pi x_{2}}{l} \right)+b_{p}sin\left( \frac{p\pi x_{2}}{l} \right) \right]\#\left( S AUTONUM \backslash* Arabic \right) \end{aligned}$$

where

$$\begin{aligned} a_{p}=\frac{1}{l}\int_{-l}^{l} H\left( x_{2} \right)cos\left( \frac{p\pi x_{2}}{l} \right)dx_{2}, p=0, 1, 2, 3,\ldots\#\left( S AUTONUM \backslash* Arabic \right) \end{aligned}$$

$$\begin{aligned} b_{p}=\frac{1}{l}\int_{-l}^{l} H\left( x_{2} \right)sin\left( \frac{p\pi x_{2}}{l} \right)dx_{2}, p=1, 2, 3,\ldots\#\left( S AUTONUM \backslash* Arabic \right) \end{aligned}$$

are called Fourier coefficients.

It should be noted that in the derivation of the one-dimensional first-order differential equation in the $x_{2}$ direction above, we only used a very limited number of terms, and each trigonometric term in $H\left( x \right)$ corresponds to two $\delta$-function subterms in the real-space $PSF$. As the number of terms increases, we can arbitrarily adjust the positions and functions of multiple frequency points that are controlled simultaneously. This also corresponds to the requirement that more complex functions need to design more complex point spread functions. In addition, the coefficient $p$ here is physically corresponding to the $\delta$-function shift distance $s_{0}$ mentioned above.


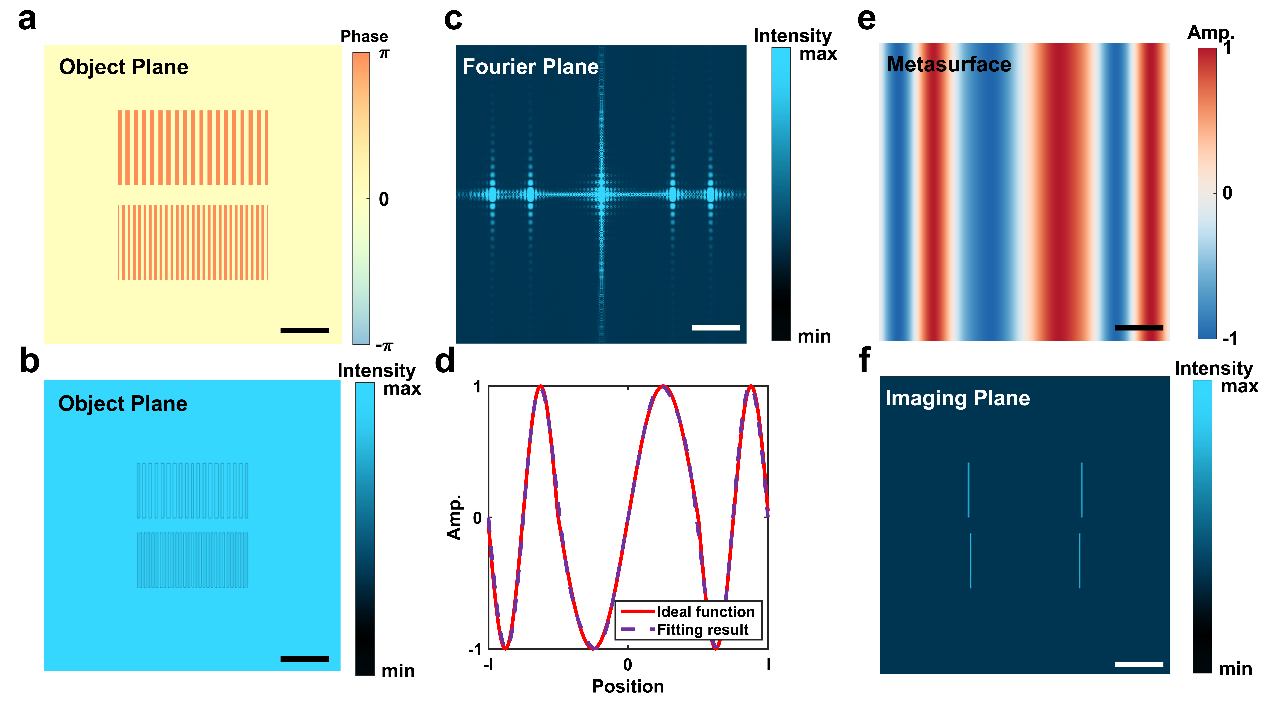


**Fig. S3: Verification of arbitrary multi-carrier signal processing. (a)** Phase information of the input image composed of two sets of phase gratings with non-integer multiple frequencies. **(b)** Intensity information of the input image, where the lines in the figure are the interference effects caused by the $\pi$ phase difference on both sides. **(c)** k-space information of the input image. **(d)** For edge extraction of the input image, the feasible modulation function and the fitting result of the triangular series expansion up to the 8th level. **(e)** Amplitude modulation obtained by loading the series expansion on the metasurface. **(f)** Output intensity result. scalebar: 500 µm.

To verify the above content, we performed a simulation of the diffraction imaging process in a 4$f$ system. As shown in **Fig. S3**, for the pure phase grating images composed of two different frequencies $f_{1}$ and $f_{2}$ ($f_{1}$ and $f_{2}$ are not in an integer multiple relationship) shown in **Fig. S3a** and **Fig. S3b**, the energy distribution in the k-space is shown in **Fig. S3c**. To achieve edge extraction of the signals on different frequency carriers, amplitude modulation shown by the red solid line in **Fig. S3d** can be used. When the series expansion reaches the 8th level, the modulation function shown by the purple dashed line in the figure can be obtained. After loading this amplitude modulation on the metasurface on the Fourier plane, the output effect shown in **Fig. S3f** can be obtained. The following contents can be seen: 1. The background intensity outside the grating is zero, which means that the plane wave background signal at the center position of the Fourier plane at $k$ = 0 is suppressed; 2. Both frequency gratings only leave edges in one direction, which means that the image signals on the two carriers $f_{1}$ and $f_{2}$ have achieved edge extraction effects.

Note 7: Arbitrary complex amplitude control with the coherent δ-function expansion approach

Though we have successfully standardized the spatial modulation of the metasurface, achieving control over arbitrary complex amplitudes remains crucial. To address this, we have implemented simultaneous modulation of both amplitude and phase by leveraging the interference between two nanobricks. In **Fig. S4**, vectors $E_{A}$ and $E_{B}$ denote the independent modulation of the two nanobricks, with vector $E_{out}$ illustrating the ultimate output signal of the metacell. Represented in polar coordinates, the vector length corresponds to amplitude modulation, while the polar angle signifies phase modulation. As depicted in the figure, we can attain coordinated adjustments of arbitrary amplitude and phase by employing two pure phase units, $E_{A}$ and $E_{B}$, whose vectors rotate on a unit circle of amplitude modulation.


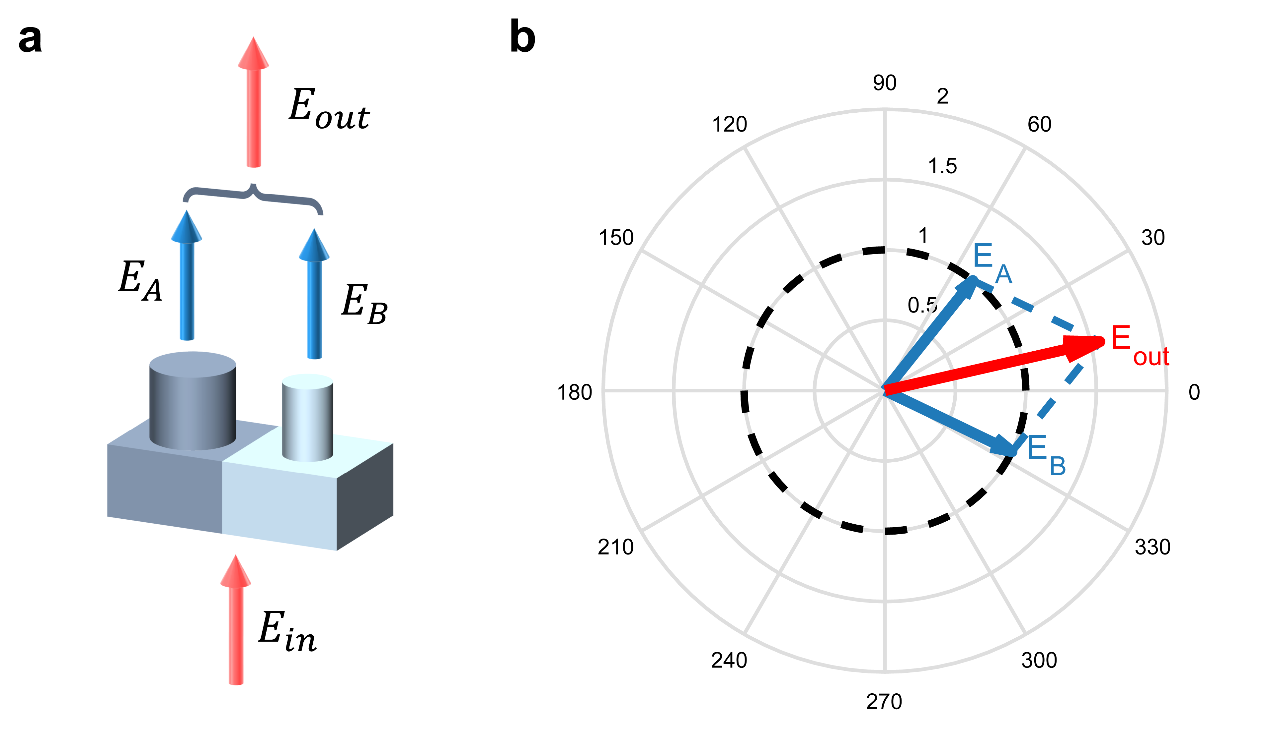


**Fig. S4: Schematic illustration of the arbitrary complex amplitude control with two neighboring metacells.**

Note 8: Additional results for the polarization multiplexing

In the polarization multiplexing experiment of **Fig. 5**, when aligning the center of the metasurface with the optical axis, two imaging targets can be separately imaged to obtain letter images showing only the edges of the graphics, as illustrated in **Fig. S5**.


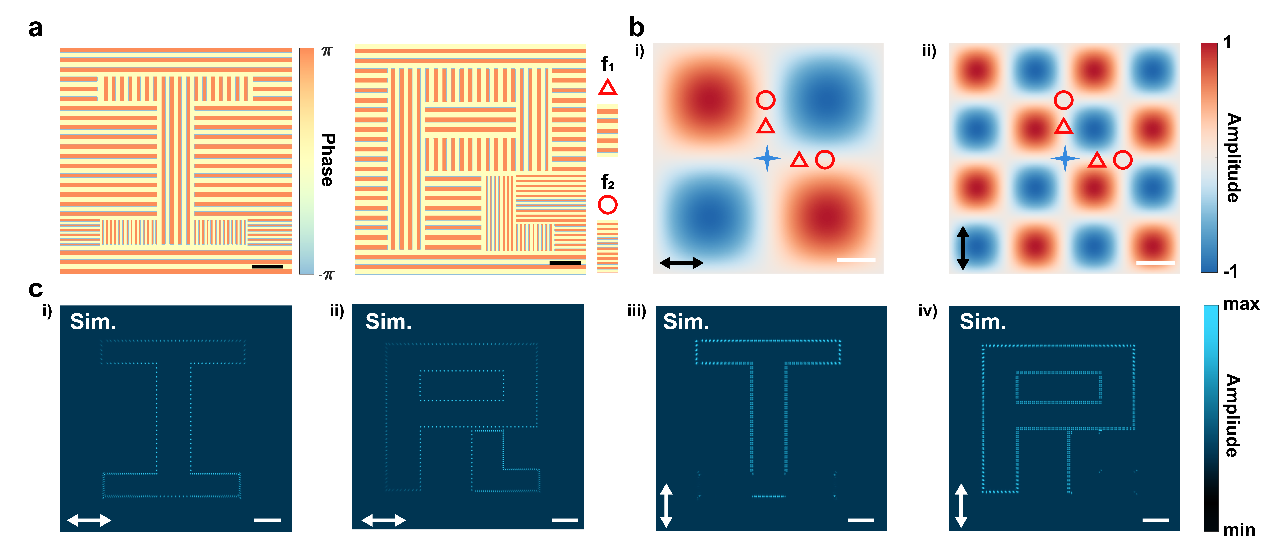


**Fig. S5: Simulation results of different images obtained when aligning the center of the metasurface with the optical axis in the experiment corresponding to Fig. 5 in the main text.** **(a)** Showcases visible light microscopy images of two uniquely fabricated phase grating targets, demonstrating the precision in manufacturing required for the modulation technique. **(b)** Details the amplitude modulation and spatial frequency mapping for: **i** $x$-polarized and **ii** $y$-polarized light. The strategic alignment of the metasurface within the Fourier plane is emphasized as critical, with the blue cross marking the metasurface’s center. Hollow triangles and circles symbolize the Fourier plane projections of the targets’ diverse spatial frequencies, illustrating the method’s nuanced control over frequency mapping. **(c)** Simulation results of different images obtained when aligning the center of the metasurface with the optical axis. Scale bar: 700 µm.

References

1. Silva A, Monticone F, Castaldi G, Galdi V, Alù A, Engheta N. Performing Mathematical Operations with Metamaterials. *Science* **343**, 160-163 (2014).

2. Arfken G, Weber H, Harris F. *Mathematical Methods for Physicists: A Comprehensive Guide* (2012).
